# The Beat

**Published:** 2010-02

**Authors:** Erin E. Dooley

## Highway Barriers Muffle Pollution

Along many heavily traveled U.S. highways stand high barriers constructed to keep traffic sights and sounds from nearby residents. An EPA/NOAA study in volume 44, issue 2 (2010) of *Atmospheric Environment* found the barriers may also help prevent air pollutants from reaching neighborhoods. The team used tracers under several different atmospheric conditions to measure the movement of pollutants such as carbon monoxide, heavy metals, and volatile organic compounds. Barriers tended to disperse or channel pollutants away from nearby areas or, in some circumstances, trap pollutants in the roadway. The authors say keeping traffic pollutants from populated areas could help reduce the incidence of respiratory illnesses, cardiovascular effects, and some cancers.

**Figure:**
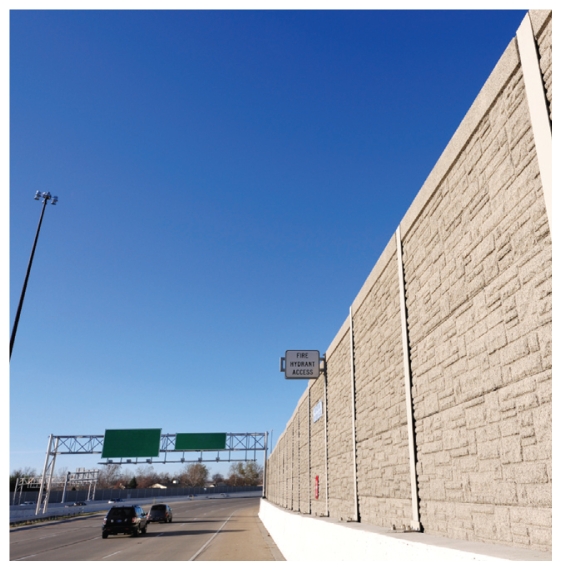
Highways barriers may contain pollutants as well as noise.

## Where There’s Smoke Flavoring . .

Smoke flavorings are used in a variety of meats, cheeses, soups, sauces, and other foods. In January 2010 the European Food Safety Authority released the results of a review showing that intake levels of 8 such flavorings may be high enough to approach levels that can cause adverse effects—although margins of safety typically overestimate intake levels. For 1 flavoring, the beech wood–derived compound AM 01, the panel could not rule out possible genotoxicity given data provided by the manufacturer. The European Commission will use the review findings to help revise the list of approved smoke flavorings.

## Methylation and Mental Retardation

Paul Greengard and colleagues report in the 10 December 2009 issue of *Neuron* that improper functioning of the protein complex GLP/G9a is linked in mice to a mental retardation–like effect known as 9q34 syndrome. GLP/G9a plays a key role in epigenetic gene silencing during normal neural development. “[I]t is conceivable,” the authors write, “that mental retardation is triggered not by changes in specific target gene(s), but by the inability of neurons to respond adequately to environmental signals under conditions of greatly distorted transcriptional homeostasis.”

## Children and Smokers: The Hazard without the Habit

In the 1 January 2010 issue of the *American Journal of Epidemiology*, Gina Lovasi and colleagues report that adults exposed to secondhand smoke in childhood may have a higher risk of emphysema-like lung damage even if they themselves never smoke. Adult participants of the MESA–Lung Study who reported living with 2 or more smokers as children were more likely to show damage on CT scans than participants who had lived with 1 or no smokers. Emphysema and chronic obstructive pulmonary disease, combined, are projected by the WHO to become the third leading cause of death worldwide by 2020.

**Figure:**
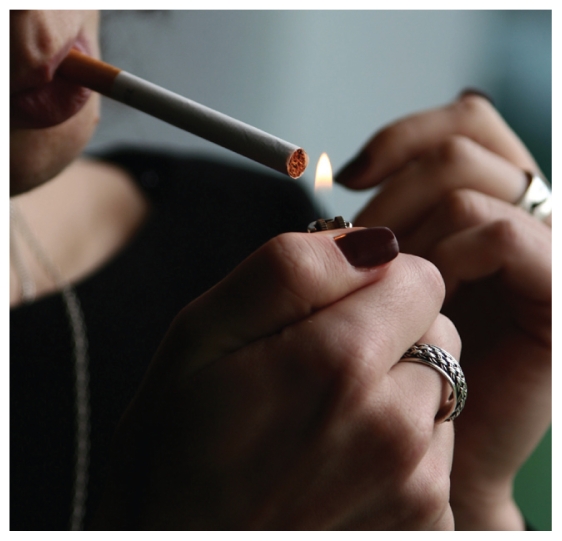
Living with multiple smokers may increase children’s risk of lung damage later even if they themselves never smoke.

## Nanotubes Detect Toxins in Water

In the December 2009 issue of *Nano Letters* Nicholas Kotov and colleagues describe a new biosensor that can rapidly detect microcystin-LR (MC-LR) in drinking water. Even small amounts of MC-LR, a peptide produced by blue-green algae, can cause liver damage and cancer, but current water treatment methods cannot always completely remove the toxin. The new biosensor consists of a paper strip containing carbon nanotubes impregnated with antibodies for MC-LR. It performs 28 times faster than the method currently used most often and produces results in less than 15 minutes. Developer Nicholas Kotov says additional toxins can be tested using their corresponding antibodies.

## Parents Take On Toy Testing

On 18 December 2009 the Consumer Product Safety Commission extended a stay of enforcement on testing for certain categories of children’s goods for up to 8 months. The extension is intended in part to work out the implementation kinks in a testing and third-party certification program originally approved in 2008. Meanwhile, even as stores scramble to remove toxic cadmium-bearing children’s jewelry from shelves, parents are taking toy safety testing into their own hands through the use of hand-held X-ray fluorescence analyzers. These devices can detect lead, cadmium, and other toxic metals in consumer products. Some health advocacy groups are purchasing the costly devices and offering their services to concerned parents during free testing events or for a fee; in some areas, the devices can also be rented.

**Figure:**
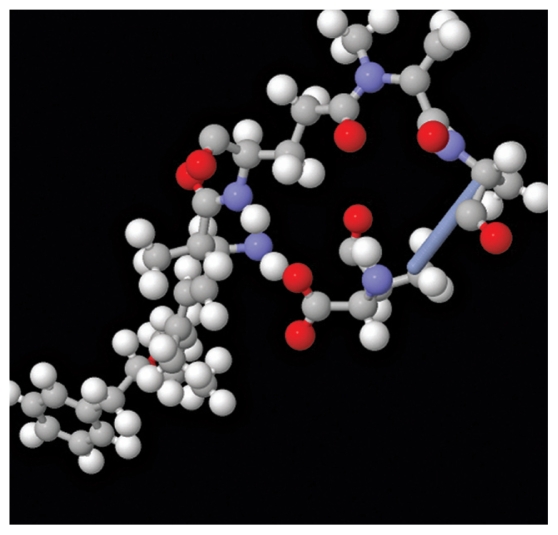
Microcyhstin-LR

